# Prevalence and associated risk factors of malaria among adults in East Shewa Zone of Oromia Regional State, Ethiopia: a cross-sectional study

**DOI:** 10.1186/s12889-017-4577-0

**Published:** 2017-07-17

**Authors:** Frew Tadesse, Andrew W. Fogarty, Wakgari Deressa

**Affiliations:** 1 School of Public Health, College of Health Sciences and Medicine, Jigjiga University, Jigjiga, Ethiopia; 2 Division of Epidemiology and Public Health, University of Nottingham, Nottingham, UK; 3 Department of Preventive Medicine, School of Public Health, College of Health Sciences, Addis Ababa University, Addis Ababa, Ethiopia

**Keywords:** Malaria, Prevalence, Diagnosis, Oromia, Ethiopia

## Abstract

**Background:**

Malaria is one of the most important causes of morbidity and mortality in sub-Saharan Africa. The disease is prevalent in over 75% of the country’s area making it the leading public health problems in the country. Information on the prevalence of malaria and its associated factors is vital to focus and improve malaria interventions.

**Methods:**

A cross-sectional study was carried out from October to November 2012 in East Shewa zone of Oromia Regional State, Ethiopia. Adults aged 16 or more years with suspected malaria attending five health centers were eligible for the study. Logistic regression models were used to examine the effect of each independent variable on risk of subsequent diagnosis of malaria.

**Results:**

Of 810 suspected adult malaria patients who participated in the study, 204 (25%) had microscopically confirmed malaria parasites. The dominant *Plasmodium* species were *P. vivax* (54%) and *P. falciparum* (45%), with mixed infection of both species in one patient. A positive microscopic result was significantly associated with being in the age group of 16 to 24 years [Adjusted Odds Ratio aOR 6.7; 95% CI: 2.3 to 19.5], 25 to 34 years [aOR 4.2; 95% CI: 1.4 to 12.4], and 35 to 44 years [aOR 3.7; 95% CI: 1.2–11.4] compared to 45 years or older; being treated at Meki health center [aOR 4.1; 95% CI: 2.4 to 7.1], being in Shashemene health center [aOR = 2.3; 95% CI: 1.5 to 4.5], and living in a rural area compared to an urban area [aOR 1.7; 95% CI: 1.1 to 2.6)].

**Conclusion:**

Malaria is an important public health problem among adults in the study area with a predominance of *P. vivax* and *P. falciparum* infection. Thus, appropriate health interventions should be implemented to prevent and control the disease.

**Electronic supplementary material:**

The online version of this article (doi:10.1186/s12889-017-4577-0) contains supplementary material, which is available to authorized users.

## Background

Ethiopia is one of the malaria-epidemic prone countries in Africa. Malaria is prevalent in over 75% of the country’s area, with 68% of the total population being at risk [1–3]. The disease was responsible for about 12% of outpatient consultations and 10% of health facility admissions, and represents the largest single cause of morbidity [4, 5]. In 2010, there were more than four million clinical and confirmed malaria cases [6]. Ethiopia is at a high risk of epidemics of malaria due to climate and topography. Broad range of epidemics happen every 5–8 years in some areas due to climatic fluctuations and drought-related nutritional emergencies [7–9]. *P. falciparum* and *P. vivax* are the two predominant malaria parasites in the country, accounting for 60–70% and 30–40% of infections, respectively [10], transmitted by inoculation by mosquitos (*Anopheles* species including *Anopheles arabiensis)* [9]*.*

As per the National Strategic Plan, the four major intervention strategies that are being applied in the country to combat malaria include early diagnosis and prompt treatment, selective vector control that involves the use of indoor residual spraying (IRS) and insecticide treated nets (ITNs) and environmental management [11]. Since 2007, malaria control interventions have been scaled-up and significantly reduced the prevalence of malaria [3]. Nevertheless, malaria is still among the leading causes of outpatient visits and hospital admissions in the country [4]. There is a scarcity of information on the prevalence of malaria among suspected malaria patients attending health facilities and the associated individual and household factors. Such information provides both a measure of the pre-test probability of a positive result as well as geographical and personal risk factors for having a positive diagnosis of infection for patients who present with symptoms of malaria. The World Health Organization (WHO) considers that when the slide positivity rate of all febrile patients with suspected malaria is less than 5%, then this region may be considered as transitioning into a state of pre-elimination of malaria infection [11].

## Methods

### Study setting and population

This study was conducted from October to November 2012 among adults aged 16 years or above who were attending five health centers in East Shewa zone of Oromia Regional State, Ethiopia. The main aim of the study was to determine the impact of health beliefs on time to presentation [12], and data were also collected on knowledge about malaria. East Shewa zone is malaria endemic area located in the Great Rift Valley in southeast Ethiopia. The climate is regarded as tropical, and this area contains a number of large lakes in a lowland setting. Based on the 2007 national census, East Shewa zone had a total population of 1,356,342; of whom 51% were men and 49% women [13]. The zone has three hospitals, 18 health centers and 296 health posts.

Malaria is the third leading cause of outpatient department (OPD) visits (36%) in East Shewa Zone. The study participants were adult patients aged 16 years or above who presented with malaria symptoms who gave blood for microscopic blood film examination at five health centers (Modjo, Meki, Batu, Bulbula, and Shashemene), each health center representing five woredas (districts). Patients who were mentally retarded, critically ill, or unwilling were excluded from the study.

### Study design and data collection

The study design was a cross-sectional study. Quantitative data were collected using pre-tested structured questionnaires, containing questions on socio-demographic characteristics, and knowledge and perception about malaria, specifically developed and applied for data collection using the local language. The interview took place after a blood sample was drawn by finger prick. The trained laboratory technicians administered the questionnaire after obtaining informed consent from an individual with malaria symptoms. The recruitment of the study participants into the study at each site sequentially continued until the required sample size for each health center was completed.

Blood was collected by experienced laboratory technicians from the finger of patients. Then smears were prepared according to the WHO protocol [14]. Parasitemia and species was determined from thick and thin smear [15], respectively. Microscopic examination of thick films using high power magnification for the presence of parasites and parasite species identification using thin films under 1000× oil immersion objective was done. A minimum of 100 consecutive fields were counted in the thick blood film before a slide was classified as negative [16].

### Statistical analysis

Data were entered using EPI INFO version 3.5.1 software package (CDC, Atlanta, GA, USA) and analyzed using SPSS version 16 (SPSS, Chicago, IL, USA) (Additional file 1). Initial analysis was done using Chi-squared test and subsequent analysis was performed by logistic regression after adjustment for potential confounding variables presented in Table 4. The data were originally collected for a study of malaria and concern about HIV testing [12] among 810 adults (16 years or above). Hence, there is no formal power calculation as this is a secondary analysis of these data. The original sample size was proportionally allocated to each health center considering the total number of suspected malaria patients tested during the previous three months **(**June–August, 2012**)** [12].

## Results

### Characteristics of the study participants

Eight hundred thirty eight individuals were approached and a total of 810 (97%) suspected malaria patients attending the health centers participated in the study, with 59% of patients from urban areas and 41% from rural areas. The median age of the patients was 27 years (ranging 16 to 80). 35% of participants had attended school to grade nine or above, while 30% had had no formal education (Table 1).

**Table 1:** Socio-demographic characteristics of the study participants

Variables	Health center					Total, *n* (%)
	Modjo	Meki	Batu	Bulbula	Shashemene	
Residence						
Rural	50	58	61	119	46	334 (41%)
Urban	119	117	119	28	93	476 (59%)
Sex						
Female	70	92	86	81	68	397 (49%)
Male	99	83	94	66	71	413 (51%)
Age						
15–24	78	46	83	48	58	313 (39%)
25–34	58	80	66	48	47	299 (37%)
35–44	24	44	24	37	13	142 (17%)
> 45	9	5	7	14	21	56 (7%)
Educational status						
No formal education	33	61	39	76	38	247 (30%)
Grade 4	28	33	26	7	9	103 (13%)
Grade 5–8	32	35	47	42	17	173 (21%)
> Grade 8	76	46	68	22	75	287 (35%)
Marital status						
Married	99	103	93	105	60	460 (57%)
Single	70	58	80	41	74	323 (40%)
Others	0	14	7	1	5	27 (3%)
Religion						
Muslim	15	44	84	125	57	325 (40%)
Christian	154	131	96	22	82	485 (60%)
Occupation						
Farmer	35	69	48	85	11	248 (31%)
House wife	25	30	26	15	24	120 (15%)
Daily laborer	27	8	24	2	6	67 (8%)
Gov. employee	21	14	16	5	17	73 (9%)
NGO employee	28	2	16	0	51	97 (12%)
Trader	5	24	6	4	3	42 (5%)
Student	28	28	44	36	27	163 (20%)
Type of roof						
Thatched	32	36	34	64	17	183 (23%)
Corrugated iron	137	139	146	83	122	627 (77%)

### Knowledge about malaria

Seven hundred ninety (97%) of the patients believed that malaria is a major health problem in the study areas. The most commonly cited malaria symptoms included feeling cold (82%), headache (76%), fever (69%), vomiting (53%), sweating (48%), and loss of appetite (49%) (Table 2). The causes of malaria were reported to be mosquito bite by 759 (94%) individuals, hunger by 276 (34%) individuals, eating maize stalk by 199 (25%) individuals, and eating immature sugar cane in 196 (24%) individuals. 803 (99%) of the patients believed that malaria is a preventable disease.

**Table 2:** Malaria knowledge and household ownership of ITNs among the study participants

Variables	Health center					Total, *n* (%)
	Modjo	Meki	Batu	Bulbula	Shashemene	
Symptoms of malaria						
Fever	151	141	97	126	46	561 (69%)
Feeling cold	143	136	131	138	117	665 (82%)
Headache	127	87	125	136	137	612 (76%)
Vomiting	81	82	105	75	84	427 (53%)
Joint pain	84	52	56	31	9	232 (29%)
Loss of appetite	114	136	66	53	32	401 (49.5%)
Muscle pain	69	20	8	10	5	112 (14%)
Nausea	85	47	11	41	50	234 (29%)
Sweating	87	134	11	37	118	387 (48%)
Malaria is preventable						
Yes	169	172	177	146	139	803 (99%)
No	0	3	3	1	0	7 (1%)
Household ownership of ITNs						
Yes	72	82	101	90	62	407 (50%)
No	97	93	79	57	77	403 (50%)
Number of ITNs owned						
1	21	28	48	25	24	146 (36%)
2	44	40	34	48	29	195 (48%)
3	7	13	12	15	7	54 (13%)
4	0	1	6	2	2	11 (3%)
Frequency of night slept under ITNs in the last 15 days						
All nights	39	28	50	56	41	214 (52%)
Sometimes	24	35	22	19	21	121 (30%)
Only few night	0	1	3	4	0	8 (2%)
None of the nights	9	18	26	11	0	64 (16%)

### Household ownership of ITNs

Fifty percent of patients with suspected malaria had any mosquito nets/ITNs in their household that can be used while sleeping. Out of those who had mosquito nets/ITNs 195 (48%) had two, 146 (36%) had only one, and 54 (13%) had three mosquito nets/ITNs. In response to a question asked about the frequency of nights slept under mosquito nets/ITNs in the last fifteen days; 214 (52%) reported all nights, 121 (30%) sometimes, and 64 (16%) none of the nights. 241 (59%) individuals reported sleeping under mosquito net/ITNs in the night prior to presentation to the health center (Table 2).

### Prevalence of malaria parasites in the study population

Two hundred four (25%) individuals in the study population had microscopically confirmed malaria parasites in their blood sample. Among those who had a positive laboratory test result, the dominant *Plasmodium* species were *P. vivax* 111 (54%), followed by *P. falciparum* 92 (45%), the remaining one (0.5%) showed mixed infections of *P. falciparum* and *P. vivax* (Table 3).

**Table 3:** Prevalence of malaria among the study participants

Variables	No. of patients	No. positive slides (%)	Positive for *P. f* (%)	Positive for *P. v* (%)
Health center				
Modjo	169	30 (18%)	16 (53%)	14 (47%)
Meki	175	70 (40%)	41 (59%)	29 (41%)
Batu	180	43 (24%)	16 (37%)	26 (60%)
Bulbula	147	19 (13%)	6 (32%)	13 (68%)
Shashemene	139	42 (30%)	13 (31%)	29 (69%)
Residence				
Rural	334	96 (29%)	47 (49%)	48 (50%)
Urban	476	108 (23%)	45 (42%)	63 (58%)
Sex				
Female	397	95 (24%)	43 (45%)	51 (54%)
Male	413	109 (26%)	49 (45%)	60 (55%)
Age				
15–24	313	93 (30%)	40 (43%)	52 (56%)
25–34	299	75 (25%)	37 (49%)	38 (51%)
35–44	142	32 (22%)	14 (44%)	18 (56%)
> 45	56	4 (7%)	1 (25%)	3 (75%)
Type of roof				
Thatched	183	56 (31%)	25 (45%)	30 (54%)
Corrugated iron	627	148 (24%)	67 (45%)	81 (55%)
Household owned at least one ITNs				
Yes	407	99 (24%)	46 (46%)	52 (52%)
No	403	105 (26%)	46 (44%)	59 (56%)
Frequency of night slept under ITNs in the last 15 days				
All nights	188	45 (24%)	20 (44%)	24 (53%)
Almost all nights	26	5 (19%)	3 (60%)	2 (40%)
Sometimes	121	31 (26%)	13 (42%)	18 (58%)
Only few night	8	2 (25%)	1 (50%)	1 (50%)
None of the nights	64	16 (25%)	9 (56%)	7 (44%)
Sought treatment before visiting the health center				
Yes	75	18 (24%)	9 (50%)	9 (50%)
No	735	186 (25%)	83 (45%)	102 (55%)
Number of days after illness onset				
≤ 2 days	140	27 (19%)	9 (33%)	18 (67%)
> 2 days	670	177 (26%)	83 (47%	93 (52%)

One individual had infection with both *Plasmodium falciparum* and *vivax*

### Factors associated with malaria positivity

Among the potential determinants explored regarding the positivity for malaria age being 16 to 24, 25 to 34, and 35 to 44 years compared to an age of 45 years or more; being in Meki or Shashemene compared to Modjo health centers; living in a rural residence compared to living in an urban area were significantly associated with positive test result for malaria. Compared to those aged 45 years or more, those who were in the age group of 16 to 24 years [Adjusted OR (aOR) = 6.7; 95% CI (2.3 to 19.5)], those who were in the age group of 25 to 34 years [aOR =4.2; 95% CI (1.4 to 12.4)], those who were in the age group of 35 to 44 years were more likely to have positive test result for malaria [aOR =3.7; 95% CI (1.2 to 11.4)] as compared to those in the age group of above 44 years. Those who were living in rural areas were more likely to have positive test result for malaria [aOR =1.7; 95% CI (1.1, 2.6)] as compared to those who were living in urban area (Table 4).

**Table 4:** Factors associated with test positivity for malaria

	Test positivity			
Variables	Negative	Positive	Crude OR (95% CI)	Adj. OR (95% CI)
Health center				
Modjo	139	30	1	1
Meki	105	70	3.1 (1.9, 5.1)	4.1 (2.4,7.1) **
Batu	137	43	1.5 (0.9, 2.5)	1.7 (0.9, 2.9)
Bulbula	128	19	0.7 (0.4, 1.3)	0.6 (0.3, 1.2)
Shashemene	97	42	2.0 (1.2, 3.4)	2.6 (1.5, 4.5)*
Residence				
Rural	238	96	1.4 (0.9, 1.9)	1.7 (1.1, 2.6)*
Urban	368	108	1	1
Sex				
Female	302	95	0.9 (0.6, 1.2)	0.8 (0.6, 1.1)
Male	304	109	1	1
Age				
15–24	220	93	6.0 (1.9, 15.6)	6.7(2.3, 19.5)*
25–34	224	75	4.4 (1.5, 12.4)	4.2(1.4, 12.4)*
35–44	110	32	3.8 (1.3, 11.3)	3.nn(1.2, 11.4)*
> 45	52	4	1	1
Type of roof				
Thatched	127	56	1.4 (0.9, 2.1)	1.5 (0.9, 2.3)
Corrugated iron	479	148	1	1
Household ownership of ITNs				
No	298	105	1.1 (0.8, 1.5)	0.9 (0.6, 1.3)
Yes	308	99	1	1
Sought treatment before visiting the health center				
Yes	57	18	0.9 (0.5, 1.6)	0.6 (0.4, 1.2)
No	549	186	1	1
Number of days after illness onset				
≤ 2 days	269	84	0.9 (0.6, 1.2)	1.3 (0.9, 1.9)
> 2 days	337	120	1	1

*Significance level of <0.05, **Significance level of <0.001

## Discussion

This study provides information regarding the prevalence of a positive diagnosis of malaria and its associated risk factors among adults with suspected malaria in malaria endemic areas located in the Great Rift Valley of southeast Ethiopia. This study has demonstrated that in a population of individuals with malaria symptoms, the prevalence of malaria was 25.2%, of which *P. vivax and P. falciparum* accounts for 54% and 45%, respectively. The present study depicts that being in the productive age group, living in Meki or Shashemene areas, and living in rural areas are risk factors for malaria infection in this population.

A significant number of *P. falciparum* cases occur in Ethiopia during the peak malaria transmission mainly in October. The national figure of 30%–40% of malaria cases in Ethiopia is due to *P. vivax* [10]. In contrast, in this study the prevalence of *P. vivax* is higher than *P. falciparum*. Likewise, *P. vivax* was the main causative agent of malaria in Oromia Regional State of Ethiopia, which accounted for 60% of slide-positive cases [3]. A study conducted in East Shewa indicated a proportion of 53% for *P. falciparum* and 47% for *P. vivax* [17]. The higher proportion of *P. vivax* in our study is consistent with studies conducted in other parts of Ethiopia [16, 18–20], which indicates trend shift of species composition. Conversely, the dominance of *P. falciparum* was indicated by other studies conducted in different parts of Ethiopia [21–23]. This could be explained by the fact that the prevention and control activities of malaria in Ethiopia [20] mainly focus on *P. falciparum* as it is deadlier than *P. vivax* [24]. Other possible reasons might be climate variability or that *P. vivax* might have developed resistance for Chloroquine.

Appropriate utilization of ITNs is one of the key interventions for the prevention of malaria [3]. In the present study, 50% of households had at least one ITN. Similarly, according to a malaria indicator survey conducted in 2011, 55% of households residing in malaria-prone areas of Ethiopia owned at least one mosquito net (of any type), and Oromia was found to have the lowest net ownership (44%) [3]. It is estimated that 42% of households in Africa owned at least one ITN in mid-2010 [25]. Moreover a study conducted in Eastern Ethiopia indicated an ITN ownership of 62% [26]. To the contrary, a study conducted in malaria epidemic prone areas of Ethiopia indicated that the overall ITN distribution was 98% [27]. The difference for this high value compared to our data could be explained by the reason that the present study is not a household survey which might have underestimated it. On the other hand, 41% of households without a single ITN represent a public health concern which needs to be addressed. The mean possession of bed net of 1.82 per household reported in our study is consistent with the report (1.73 /household) from study conducted in Ghana [28]. However, it is by far higher than the findings of malaria indicator survey conducted in 2011 (mean 0.7 /household) [3]. The ITN utilization of our study is high as compared to the study conducted in Eastern Ethiopia (21.5%) [26].

The use of representative samples with a high response rate of 97% is the strength of the present study, however it has some limitations. This study is a facility based survey. Therefore, it does not represent the situation in the whole population but it already provides reliable important data. Data collection relied on information given by the interviewees. Practices such as presence, type and use of ITN could not be verified by direct observation. Moreover, the diagnosis of malaria did not include PCR (Polymerase Chain Reaction). As this was a pragmatic study in a real-life rural environment, blood film was available to diagnose malaria infection, rather than rapid diagnostic testing which has a higher sensitivity [29]. On top of that, microscopic tests of malaria were done by the laboratory technicians in the different settings who didn’t get training about the determination of test positivity which could have led to bias due to interpersonal variation. However, these details reflect the ‘real-world’ nature of our data, that were based on usual clinical practice, and do not necessarily invalidate our findings.

## Conclusions

In conclusion, findings of this study indicate that malaria is an important public health problem among adults in East Shewa with the predominance of *P. vivax* and *P. falciparum*; and being in the productive age group, living in Meki or Shashemene, and living in rural areas, were risk factors for malaria infection. According to WHO when the slide positivity rate of all febrile patients with suspected malaria is less than 5%, the country could consider transitioning into “pre-elimination” [11]. Therefore, a test positivity rate of 25% at health facility level indicates that malaria is a major burden in the zone, which is not in line with the national strategic plan for malaria prevention control and elimination in Ethiopia. Moreover, there is a gap regarding the mosquito nets/ITNs ownership and utilization. Hence, more focus should be given to environmental sanitation as well as the consistent utilization of ITNs should be promoted by health workers and health extension workers in particular. In addition, the number of mosquito nets/ITNs supplied to households should be increased in order to assure adequate mosquito nets/ITNs ownership in each household. Further study using direct observation at sleeping time rather than reported use is important to assess ownership proper utilization of ITNs. Special attention should be given to those living in the rural area of the zone. Furthermore, there was an increased risk of malaria infection among the younger age group as well as among those living in Meki and Shashemene areas which needs a further investigation.

## Additional file

Additional file 1: SPSS data set for the research entitled "Prevalence and associated risk factors of malaria among adults in East Shewa Zone of Oromia Regional State, Ethiopia: A cross-sectional study". (SAV 637 kb)

## Abbreviations

IRS: Indoor Residual Spraying
ITN: Insecticide Treated Net
OPD: Outpatient Department
PCR: Polymerase Chain Reaction
WHO: World Health Organization
